# Larvoscopic study on *Dictyocaulus* sp. in the faeces of beef cattle in northeastern Brazil

**DOI:** 10.1590/S1984-29612022047

**Published:** 2022-08-19

**Authors:** Lucia Oliveira de Macedo, Carlos Roberto Cruz Ubirajara, Renata Silva Brito, Karlla Keyla Ferreira dos Santos, Carla Lopes de Mendonça, Gílcia Aparecida de Carvalho, Rafael Antonio Nascimento Ramos

**Affiliations:** 1 Laboratório de Parasitologia, Universidade Federal do Agreste de Pernambuco, Garanhuns, PE, Brasil; 2 Programa de Pós-graduação em Biociência Animal, Universidade Federal Rural de Pernambuco, Recife, PE, Brasil; 3 Clínica de Bovinos de Garanhuns, Universidade Federal Rural de Pernambuco, Garanhuns, PE, Brasil

**Keywords:** Nematode, lungworm, epidemiology, livestock production, Nematódeo, verme pulmonar, epidemiologia, produção pecuária

## Abstract

The lungworm *Dictyocaulus viviparus* has an important role in cattle health and productivity worldwide, since infections can lead to substantial economic losses. Despite its importance, few studies investigating the epidemiological aspects of infection by this parasite have been conducted. The aim of this study was to report the occurrence of lungworm infection in beef cattle herds reared in an area of livestock production in the northeastern region of Brazil. From September 2020 to August 2021, monthly fecal samples (*n* = 493) were collected from 46 beef cattle. Among all the animals assessed, lungworm larvae were detected in 8.7% (4/46). None of them presented any clinical sign suggestive of infection by lungworm parasites. Twenty larvae were retrieved, with the minimum number (*n* = 1) detected in October and December, and the maximum number (*n* = 13) in November. These presented a mean length of 363 μm (± 28.65 μm) and mean width of 19 μm (± 1.03 μm), and were morphologically similar to *Dictyocaulus* sp.. This study reports the occurrence of this parasite in this livestock production area. Lastly, local veterinarians need to be aware of inclusion of this parasite in the differential diagnosis of other respiratory infections in beef cattle.

## Resumo

O parasito pulmonar *Dictyocaulus viviparus* tem um papel importante na saúde e produtividade bovina em todo o mundo, uma vez que infecções podem levar a perdas econômicas. Além de sua importância, poucos estudos que investigam aspectos epidemiológicos das infecções por esse parasito têm sido realizados. Objetivou-se, nesta pesquisa, relatar a ocorrência de infecção por nematódeos pulmonares em rebanhos bovinos de corte, criados em uma importante área de produção pecuária, na região Nordeste do Brasil. De setembro de 2020 a agosto de 2021, foram coletadas, mensalmente, amostras fecais (n = 493) de 46 bovinos de corte. De todos os animais avaliados, larvas de nematódeos pulmonares foram detectadas em 8,7% (4/46). Os animais não apresentaram nenhum sinal clínico sugestivo de infecção por parasitos pulmonares. Vinte larvas foram recuperadas, com o número mínimo (n = 1) detectado em outubro e dezembro, e o número máximo (n = 13) em novembro. Apresentavam comprimento médio de 363 μm (± 28,65 μm), largura média de 19 μm (±1,03 μm) e eram morfologicamente semelhantes a *Dictyocaulus* sp.. Este estudo relata a ocorrência desse parasito nesta área. Logo, os veterinários locais devem estar atentos à inclusão desse parasito no diagnóstico diferencial de outras infecções respiratórias em bovinos de corte.

Infections by lungworm parasites play an important role in ruminants’ health worldwide (Kuchboev et al., 2012; Verocai et al., 2020; Macedo et al., 2021). In cattle, this infection is manifested as parasitic bronchitis (PB) and is caused especially by the lungworm species *Dictyocaulus viviparus* (Nematoda: Dictyocaulidae). This nematode has been considered to be economically important for these animals at different stages of their life (Forbes, 2018). It has a direct life cycle and cattle become infected in pastures contaminated with third-stage larvae (L3) (Jorgensen, 1980). After infection, this parasite colonizes the lower respiratory tract of cattle, where females lay eggs that quickly hatch after release. The first-stage larvae are then swallowed and eliminated in feces (Panuska, 2006). Infection with these nematodes can lead to subclinical or severe clinical disease with clinical signs like coughing and dyspnea, and may eventually lead to the host’s death (Ploeger et al., 2012; Holzhauer et al., 2011; May et al., 2018).

For a long time, PB caused by *D. viviparus* was considered to be of clinical relevance mainly during the first year of calves’ lives. However, more recently, outbreaks of PB have been reported in adult herds, with considerable economic losses, due to reduction of productivity and due to mortality (Holzhauer et al., 2011). For example, in an outbreak reported in the Netherlands, it was demonstrated that the loss in milk production could reach 4 kg/cow/day (Holzhauer et al., 2011). On the other hand, in Belgium, it was shown that animals with subclinical infection had a mean loss of milk production of about 0.5 kg/cow/day (Charlier et al., 2016). Unfortunately, this economic loss may be underestimated, especially when mortality occurs, as was reported from a beef cattle herd in Malaysia (Lat-Lat et al., 2007).

In Brazil, there have only been reports of infection by *D. viviparus* in the southern and southeastern regions (Landim et al., 2001; Henker et al., 2017; Cezaro et al., 2018; Schade et al., 2020). In most studies, the occurrence was reported at postmortem examinations (Gonçalves et al., 2000; Silva et al., 2005). Although few data are available from the northeastern region, there are personal communications from veterinarians reporting clinical cases of respiratory disease suggestive of lungworm infection in cattle (Macedo et al., 2021).

The classic method for diagnosing lungworm infection in cattle is the Baermann technique, which is based on retrieval and morphological identification of larvae in fresh fecal samples (Forrester & Lankester, 1997). However, over time, greater attention has been given to gastrointestinal parasites and this method has been little applied, especially in relation to cattle without clinical signs of PB (May et al., 2018). Additionally, the different levels of sensitivity of the Baermann method, according to the age of the animals, has hampered real knowledge about the rate of occurrence of *D. viviparus* in cattle worldwide (Charlier et al., 2016).

Therefore, the aim of the present study was to report the occurrence of lungworm infection in beef cattle herds reared in an important area of livestock production in the northeastern region of Brazil.

This study was conducted on a beef cattle farm (Nellore breed), located in the municipality of Quipapá (8º83’64” South and 36º09’30” West), in the Zona da Mata region of the state of Pernambuco, northeastern Brazil. This area is characterized by a hot and humid tropical climate (*As*), with an average annual temperature of around of 23.4 to 25.8 °C, an annual mean rainfall of 400 mm to 900 mm and air relative humidity of 90% (Medeiros et al., 2021).

The Ethics Committee for Animal Experimentation (ECAE) of the Federal Rural University of Pernambuco (*Universidade Federal Rural de Pernambuco*) approved all the procedures performed in this study (approval number: 21/2019).

In July 2020, two animals belonging to a herd of 48 animals (ranging in age from 5 to 8 months; 6 males and 40 females) were attended at the Bovine Clinic of Garanhuns (Federal Rural University of Pernambuco). These two animals died, and at postmortem examination numerous filiform helminths (not identified morphologically) were observed in the trachea and bronchi. The animals did not present any clinical signs suggestive of infection by lungworm parasites and, thus, the detection of nematodes was a finding.

After this event, the herd (n = 46) was monitored monthly and fecal samples from each animal were collected over a period of 12 months (from September 2020 to August 2021). Feces were collected directly from the rectum using plastic gloves. They were kept in isothermal boxes (8 ºC) and processed in until 6 hours after collection. The herd was fed on *Panicum maximum* pasture, with supplementation consisting of corn silage and concentrate feed. The meteorological data (temperature, relative humidity and rainfall) were obtained from the National Institute of Meteorology (INMET).

Fresh fecal samples were individually processed using the Baermann technique (Forrester & Lankester, 1997). The larvae found were morphologically analyzed and features of their anterior and posterior ends were recorded (Soulsby, 1968). Measurements were obtained using the software TCapture 4.3.

Descriptive statistical analysis was performed to obtain relative and absolute frequencies of positivity. Exact binominal 95% confidence intervals (CIs) of Wilson scores were established as proportions. The chi-square statistic with Yates correction was used to compare proportions, and probability *p* values < 0.05 were regarded as statistically significant. All the analyses were performed using the Epitools epidemiological and chi-square calculators.

A total of 493 samples from 46 animals were analyzed during the whole study period, with a mean of 41 ± 4 animals assessed per month. Out of all the samples analyzed, lungworm larvae were detected in 1.4% (7/493; 95% CI = 0.69-2.90). Among the animals, 8.7% (4/46; 95% CI = 0.34-20.32), comprising two males and two females, were positive (*p* = 2.3103; χ^2^ = 0.1285). During the entire study period, these animals did not present any clinical sign suggestive of infection by lungworm parasites.

**Figure 1:**
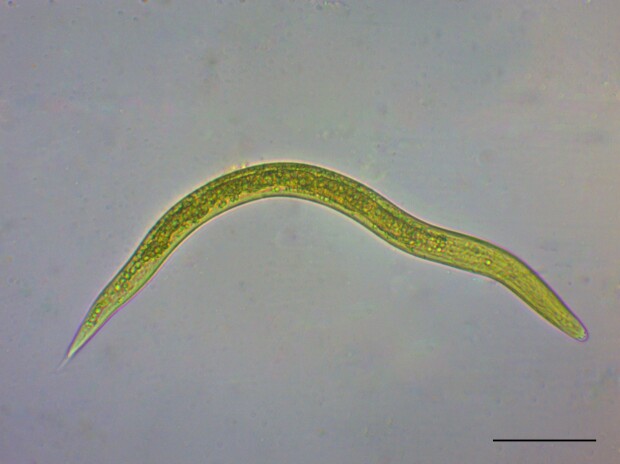
First-stage larva (L1) of *Dictyocaulus* sp. detected in feces from cattle in northeastern Brazil (scale-bar = 50 μm).

A total of 20 larvae were retrieved, with the minimum number (*n* = 1) detected in October and December, and the maximum number (*n* = 13) in November. These presented a mean length of 363 μm (± 28.65 μm) and mean width of 19 μm (±1.03 μm). In addition, morphologically, they were similar to *Dictyocaulus* sp., with intestinal cells containing numerous granules, typical of the family Dictyocaulidae (Figure 1).

**Figure 2:**
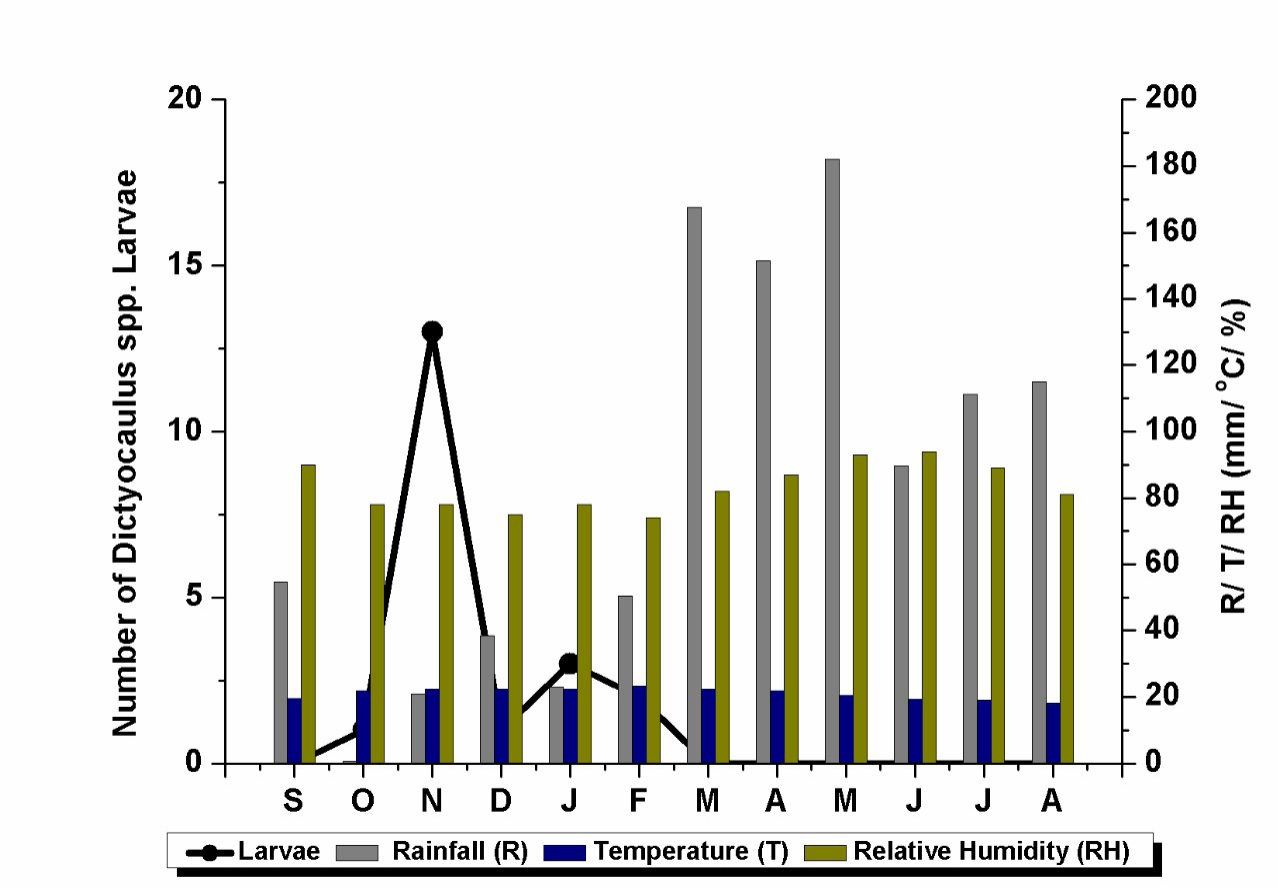
Overall distribution of larvae during the study period according to the climatic data.

The overall distribution of larvae during the study period according to the climatic data is reported in Figure 2.

This study assessed the occurrence of *Dictyocaulus* sp. infection in beef cattle herds reared in an important area of livestock production in the northeastern region of Brazil. Until now, occurrences of this parasite had only been reported in the southeastern and southern regions of this country (Landim et al., 2001; Henker et al., 2017; Cezaro et al., 2018). A previous study reported an outbreak with morbidity and lethality rates of 7.1% and 13.3%, respectively, in young animals in the state of Rio Grande do Sul (Silva et al., 2005).

Similarly, to what we report here, a study in Costa Rica found that the prevalence of infection (1.8%; 9/549) remained low throughout the study period (Jiménez et al., 2007). It is important to note that the common practice of administration of anthelmintics was also adopted in the farm of the present study. On this farm, the anthelmintic drug ivermectin was applied every three months, specifically in November, February and May. Undoubtedly, this gave rise to difficulty in retrieving lungworm larvae from the animals of this study.

Our results indicated that males and females (*p* = 3.206; χ^2^ = 0.733) were equally affected, although out of the six males present in the herd, two were infected. Previous studies on small ruminants have also shown that males are commonly more affected, thus suggesting that the different types of nutrition among these animals can influence lungworm infection (Borji et al., 2012). However, it was difficult to detect any effects of sex on the prevalence of lungworm infection because of the large difference in the numbers of males (n = 6) and females (n = 40) assessed in this study.

Infection by *Dictyocaulus* sp. in cattle is often suspected due to clinical signs such as coughing and increased respiratory rate (Ploeger et al., 2012; May et al., 2018). Nevertheless, over the entire study period, none of the animals of the present study showed any clinical sign suggestive of infection by lungworms. It is believed that cattle develop protective immunity and therefore do not develop any clinical disease (May et al., 2018).

Although the prevalence detected here was relatively low (i.e., 8.7%), presence of lungworms was detected in five consecutive months (October 2020 to February 2021). A previous study in the southeastern region of Brazil demonstrated that the prevalence of *D. viviparus* were 50%, 35% and 28.5%, in the autumn, winter and summer, respectively (Cezaro et al., 2018). The larval count in the present study ranged from one to seven larvae per 40 g of feces for each animal, which was in accordance with previous studies (May et al., 2018). However, we cannot discard the possibility of false negative results, especially because the Baermann technique presents high sensitivity (100%) only for detection of primary infection in young animals (Eysker, 1997).

In Brazil, reports using the Baermann technique to detect this lungworm species are sparse. Most records have been obtained through postmortem examinations, although there is a relatively simple in vivo diagnostic test that can be implemented (Verocai et al., 2020). For example, a recent retrospective epidemiological study covering the last four decades showed that only 20.8% of the animals were diagnosed using the Baermann technique on live animals (Macedo et al., 2021). In general, lungworm infection in ruminants may be of minor importance, compared with gastrointestinal parasites, and is therefore largely underdiagnosed, which leave gaps in the knowledge of the distribution and epidemiology of these parasites. Even if the absence of molecular analysis had been considered an important limitation of this study, the morphological identification of larvae in animals from a herd with a previous historic of infection by lungworm indicates the presence of these parasites in this area. Undoubtedly, molecular data will be addressed in the future to determine the parasite at level species.

Lastly, the data presented here demonstrated the rate of occurrence of *Dictyocaulus* sp. in fecal samples collected from beef cattle in Northeastern Brazil. Although no clinical signs were observed among these animals, veterinarians and farmers need to be aware of the importance of this parasite in the differential diagnosis of other respiratory infections, such as viral and bacterial infections. Knowledge of this kind of infection in this region will be useful for guiding decisions regarding anthelmintic treatments and other control strategies to prevent clinical disease and mortality, and subsequently to minimize potential economic losses.
